# Measurement of Electrical Discharge Machining Oil Quality by Analyzing Variations in the Equivalent Relative Permittivity of the Capacitive Sensor

**DOI:** 10.3390/s20216248

**Published:** 2020-11-02

**Authors:** Shih-Jui Chen, Yi-Li Chen, Yu-Jui Chang, Dong-Lin Chuang, Yi-Chun Chen, Hai-Ping Tsui, Yean-Ren Hwang

**Affiliations:** 1 Department of Mechanical Engineering, National Central University, Taoyuan 32001, Taiwan; leo831126@hotmail.com (Y.-L.C.); yrchang@g.ncu.edu.tw (Y.-J.C.); chuangdonglin@gmail.com (D.-L.C.); benno@ncu.edu.tw (H.-P.T.); yhwang@cc.ncu.edu.tw (Y.-R.H.); 2 Department of Optics and Photonics, National Central University, Taoyuan 32001, Taiwan; ycchen@dop.ncu.edu.tw

**Keywords:** capacitive sensors, electrical discharge machining oil, equivalent relative permittivity

## Abstract

In this study, a concentration monitoring system was successfully developed. A sensor was immersed in electrical discharge machining oil, and the capacitance of the sensor changed as a function of the impurity concentration. Thus, DC voltage variations were produced via a conversion circuit. Carbon black and iron particles with different concentrations were successfully characterized. The capacitance increments were positively correlated with the particle concentration. The linear fitting results based on the impurity concentration were used to express the degree of influence of particles with different weight percentage concentrations on the increase in the overall capacitance value. An equivalent medium theory model was then developed according to the electrical characteristics of the impurities to predict different particle volume percentages.

## 1. Introduction

Electrical discharge machining (EDM) is a process in which sparks are generated by discharge, and material removal is achieved through repeated discharges between the workpiece and electrode. A high discharge energy produces high temperatures at the spark point during processing, which causes the workpiece to melt and evaporate. The processed workpiece is flushed with kerosene-based insulating processing oil to remove workpiece debris. If the melted material is not washed away, the workpiece surface is covered with a resolidified layer and becomes rough. The presence of a considerable amount of debris is believed to cause arcing sparks, which result in an unstable and inefficient process [1,2,3].

In the filter blockage method, when oil passes through the microporous filter, particles of different concentrations in the oil accumulate and gradually block it, causing a pressure difference or flow change at both ends. Lu [4] designed a metal membrane filter to measure the degree of oil contamination by analyzing the weight concentration of particles. This method is based upon particle size, and the fabrication of the filter involves a delicate process.

The electrical measurement method involves the determination of the electrical conductivity or dielectric constant of the oil when the particle concentration changes. When a change in the characteristic impedance is detected, the degree of contamination of the oil can be determined.

In the resistive detection method, when metal particles are generated in oil, the electrical resistivity of the oil decreases; therefore, metal debris can be monitored by measuring oil resistance. Itomi [5] developed a metal powder sensor to detect the presence of iron filings in oil. However, measuring the electrical resistance of oils with a low detrital concentration is difficult.

Raadnui [6] developed a grid capacitance sensor to detect variations in lubricant quality caused by contaminants. Dervos [7] designed a cylindrical sensor for permittivity characterizations of high-voltage transformer oils. Nezami [8] developed a capacitive sensor to sense 2-FAL concentration in the transformer oil by dip coating a molecularly imprinted polymer on electrodes. Shi [9] developed an inductive-capacitive sensor to detect debris in hydraulic oil in order to determine the working state of hydraulic systems. Murali [10] designed a monitoring microchannel device to determine the degree of contamination of processing oil. When aluminum metal debris passes through the microchannel, the change in capacitance can be detected. Khaled [11] used a capacitive sensor with an interdigitated electrode structure to measure the deterioration of frying oil.

In this study, a dynamic capacitive sensor that measures the concentration of carbon black and iron particles in an EDM machine was developed. When measurements are performed for the EDM process, the amount of debris generated by machining increases with machining time, which increases the turbidity of the processing oil. At this time, the voltage output value measured by the sensor also increases, and the current degree of contamination can be obtained from the equivalent dielectric constant of the processing oil.

## 2. Theory

### 2.1. Measurement of the Dielectric Constant from the Impedance of the Capacitor

During EDM, workpiece scraps are generated when a high amount of energy is used for material removal. For example, in this study, SS41 low-carbon steel was used as the processing piece, which produced iron scraps during EDM. If the hydrocarbon base oil is incompletely burned, soot is generated. These impurities are randomly dispersed in the processing oil. Therefore, this research aimed to examine the processing oil through a capacitive sensor. Typically, an AC signal is used to test the dielectric properties of a material, and the dielectric constant is determined by measuring the impedance of the capacitor.

If a medium enters a parallel-plate capacitance sensor, its dielectric constant $\varepsilon_{r}$ in the case of AC is usually expressed as a complex term as follows [12,13,14]:

(1) $$\varepsilon_{r}=\varepsilon_{r}^{\prime }-j\varepsilon_{r}^{\prime \prime }=\varepsilon_{r}^{\prime }-j\frac{\sigma_{DC}}{\omega \varepsilon_{0}},$$

where $\sigma_{DC}$ is the DC conductivity and ω is the angular frequency. By substituting Equation (1) into the capacitive impedance $Z_{c}$, the following equation is obtained:

(2) $$Z_{c}=\frac{d}{j\omega \varepsilon_{0}A\left(\varepsilon_{r}^{\prime }-j\varepsilon_{r}^{\prime \prime }\right)},$$

where d is the gap between the parallel plates and A is the area of the parallel plates.

The behavior of dielectrics under a specific alternating field can be described using an equivalent RC parallel circuit model. The dielectric experiences energy loss in the generated AC field [15]. For the convenience of calculation, the impedance can be written as an admittance as follows:

(3) $$Y_{c}=\frac{1}{Z_{c}}=\frac{j\omega \varepsilon_{0}A\varepsilon_{r}^{\prime }}{d}+\frac{\omega \varepsilon_{0}A\varepsilon_{r}^{\prime \prime }}{d}\;=j\omega C+G,$$

where the susceptance is ωC, conductance is G, $\varepsilon_{0}$ is the permittivity in a vacuum, and $\varepsilon_{r}^{\prime }$ is the real part of the dielectric constant for calculating the capacitance C, as shown in Equation (4).

(4) $$C=\frac{\varepsilon_{0}A\varepsilon_{r}^{\prime }}{d}.$$

The main substance to be measured in this study is a hydrocarbon solvent. The literature [16,17] indicates that nonpolar substances include insulating oils, such as transformer oil or other hydrocarbon lubricating oils. The capacitance of such nonpolar substances is unaffected by the frequency and resembles a static dielectric constant. Although the conductance increases with increasing frequency, the energy loss $\varepsilon_{oil}^{\prime \prime }$ of the dielectric at low frequencies is negligible. For pure oils, $\varepsilon_{oil}^{\prime }$ remains stable. Therefore, this study reasonably approximated $\varepsilon_{oil}^{\prime \prime }$ to $\varepsilon_{oil}$ and did not consider the imaginary part of the complex permittivity under a low-frequency AC signal of 10 kHz.

When changes in the capacitance values of the medium to be tested and the air medium are measured, the dielectric constant of the medium to be tested can be derived as follows:

(5) $$\varepsilon_{r}^{\prime }=\frac{d}{\varepsilon_{0}A}\left(C-C_{air}\right)+1,$$

where $C_{air}$ is the capacitance of air.

### 2.2. Equivalent Medium Theory Model

**Figure 1:**
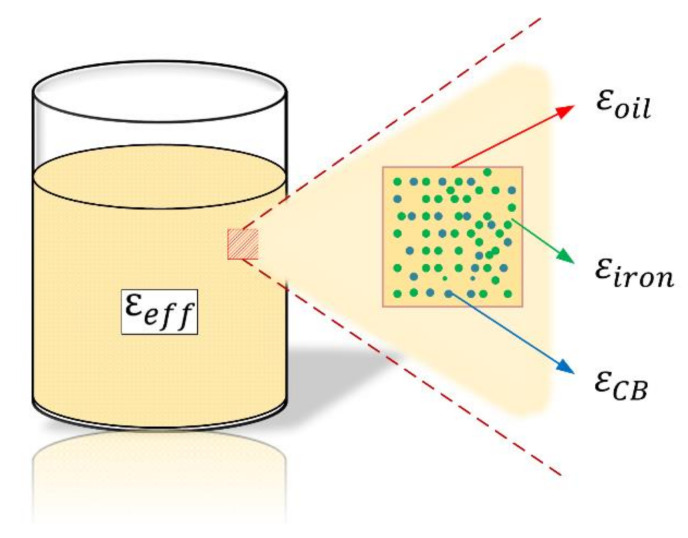
Schematic of randomly dispersed impurities in processing oil.

Figure 1 depicts a schematic of the mixing of heterogeneous materials. During EDM, the processing debris is randomly dispersed into processing oils that have different dielectric constants, and the mixed mass has an equivalent dielectric constant $\varepsilon_{eff}$.

In this study, the carbon black and iron filings generated during EDM were highly conductive. For example, the carbon black usually has a direct current conductivity ($\sigma_{DC}$) of $3.5\;\times \;10^{2}$ S/m [18]. Iron has a $\sigma_{DC}$ value of approximately $10^{7}$ S/m [15]. The dispersion of electrically conductive materials in nonpolar insulating processing oils may result in an increase in the overall dielectric constant. In the field of composite materials, many studies have embedded conductive fillers into the insulating matrix. This method has been shown to improve the electromagnetic frequency interference shields and electrical conductivity. For example, carbon black polymer [19], Fe–Al_2_O_3_ [20], and aluminum epoxy [21] are composite materials with conductive components in the insulating matrix.

To effectively predict the equivalent dielectric constant of impure processing oils, this study referred to the equivalent medium theory (EMT) model and method used in [19]. The dielectric constant $\varepsilon_{filler}$ for the conductive filler can be approximated as follows: $-j{\varepsilon^{\prime \prime }}_{filler}=-j\frac{\sigma_{DC,filler}}{\omega \varepsilon_{0}}$ and $\left|{\varepsilon^{\prime }}_{filler}\right|\ll {\varepsilon^{\prime \prime }}_{filler}$.

Most dielectric materials exhibit partial energy loss at alternating frequencies, and these materials are not perfect insulators. The relationship between the conductivity and the complex dielectric function can be obtained from (1). However, for materials with high electrical conductivity, the complex dielectric function is made up, for the most part, by the imaginary part. Therefore, the complex dielectric function can be reasonably approximated as the imaginary part [19]. In addition, some studies regard the complex dielectric constant of metal conductors to be infinite [21,22]. According to the optical characteristics and Drude model, the real part of the complex dielectric function of a metal is negative at low frequencies (usually below the frequency of visible light).

EMT has been adopted in many studies, and different types of mixed formulas have been examined [19,21,22,23,24]. In this study, we used the following mixture formula:

(6) $${\varepsilon_{eff}}^{\beta }=V_{oil}{\varepsilon_{oil}}^{\beta }+V_{CB}{\varepsilon_{CB}}^{\beta }+V_{iron}{\varepsilon_{iron}}^{\beta },$$

where $\varepsilon_{eff}$ is the equivalent dielectric constant of the mixed medium; $\varepsilon_{oil}$ is the dielectric constant of pure oil; $\varepsilon_{CB}$ and $\varepsilon_{iron}$ are the dielectric constants of the carbon black (CB) and iron, respectively; $V_{CB}$ and $V_{iron}$ are the volume fractions of the CB and iron, respectively; and β is a fitting parameter whose value depends on the type of mixture.

## 3. Transducer System

**Figure 2:**
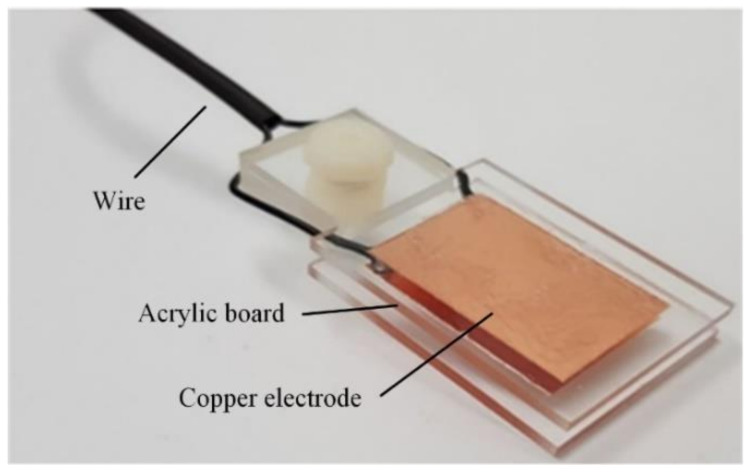
Photo of the sensor structure.

The adopted sensor comprises a copper electrode and a sensing circuit. The sensor was made of transparent acrylic. The capacitor depicted in Figure 2 was made of 35-µm-thick copper. A 2 × 3 cm^2^ electrode was adhered to two acrylic boards separated by 3 mm. When the parallel-plate capacitive sensor was immersed in different concentrations of oil, different polarization under the action of the electric field occurred. To make the potential the same as when there is no dielectric, more charges must be provided.

**Figure 3:**
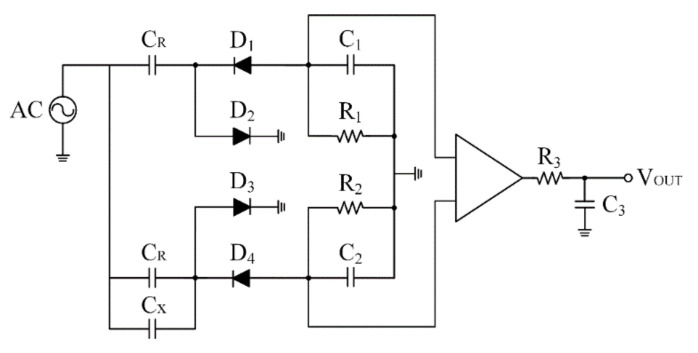
Schematic of the adopted circuit.

The sensing circuit is described in Figure 3. The capacitance signal of the sensor was input into an AC bridge conversion circuit. Then, an amplifier and a filter were used to obtain a DC voltage, which was utilized to measure the signal change of the sensor. The capacitance of the processing oil with different capacitance ranges can be measured by adjusting the circuit parameters. The change in capacitance can be converted to the real part of the relative complex permittivity using Equation (5).

In Figure 3, C_R_ is the reference capacitance, and C_X_ is the capacitance to be measured. We tested the relationship between the output DC voltage and the measured capacitance using standard capacitors. The sensor capacitance was positively correlated with the output voltage. The sensitivity of the circuit was 0.475 V/pF.

## 4. Experimental Setup

**Figure 4:**
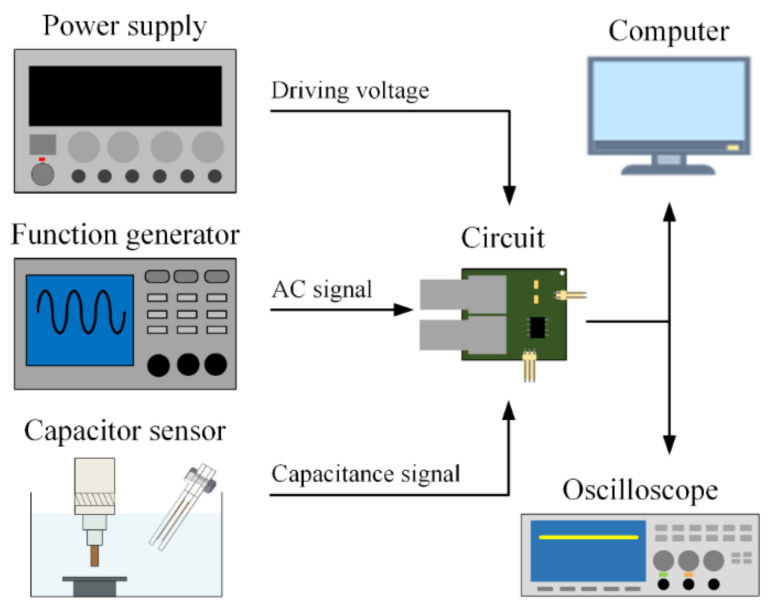
Schematic of the experimental setup.

The experimental setup is displayed in Figure 4. First, the processing oil enters the capacitive sensor through a flow path. When the medium between the two electrode plates changes, the sensor capacitance changes and is converted into a DC voltage. Finally, the measurement results can be observed using an oscilloscope and recorded by a computer for comparison. The real-time change in the processing oil concentration during EDM was also monitored.

## 5. Results and Discussion

Common materials in EDM are carbon black and metal scrap. Therefore, the measurement part was divided into three parts: the measurement of carbon black, the measurement of iron particles, and the real-time measurement during EDM. The sensing range of carbon black and iron particles was set between 0~3 g/100 mL, since the corresponding capacitance variation for machining SS41 low-carbon steel was within this range.

### 5.1. Capacitance Measurement for Carbon Black Powder

**Figure 5:**
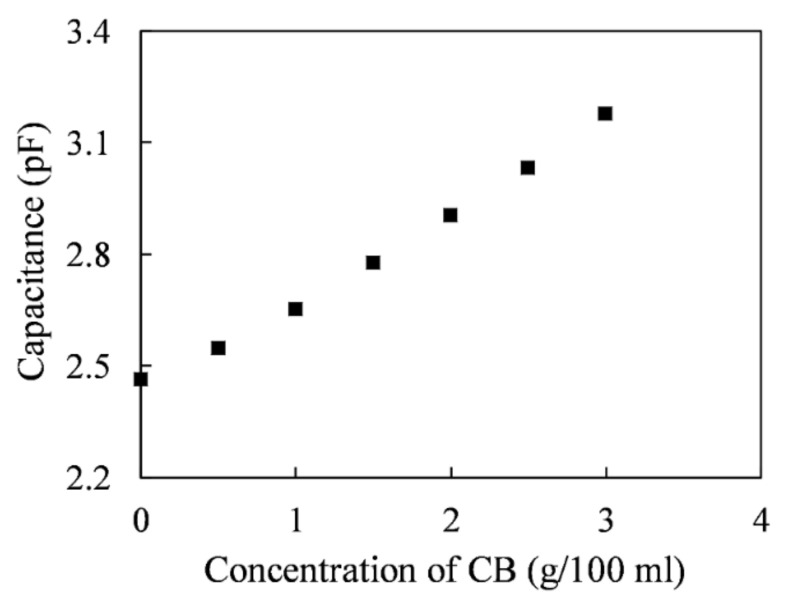
Measured capacitance increment of the carbon black.

The capacitances of carbon black powders at each concentration are plotted in Figure 5. The capacitances were measured by adding different weights of carbon black to 100 mL of EDM oil. As the concentration of the carbon black particles increased, the measured capacitance increased. The initial capacitance value is 2.46 pF. The capacitance of carbon black was 2.55 pF at a concentration of 0.5 g/100 mL. The capacitance increased to 3.18 pF when the concentration increased to 3 g/100 mL.

Therefore, the average capacitance increment for carbon black was 0.24 pF per 1 g/100 mL variation in concentration. The higher the amount of carbon black in the processing oil, the higher was the output voltage of the capacitive sensor. Thus, the equivalent dielectric constant increased with the carbon black concentration.

### 5.2. Capacitance Measurement for Iron Powder

**Figure 6:**
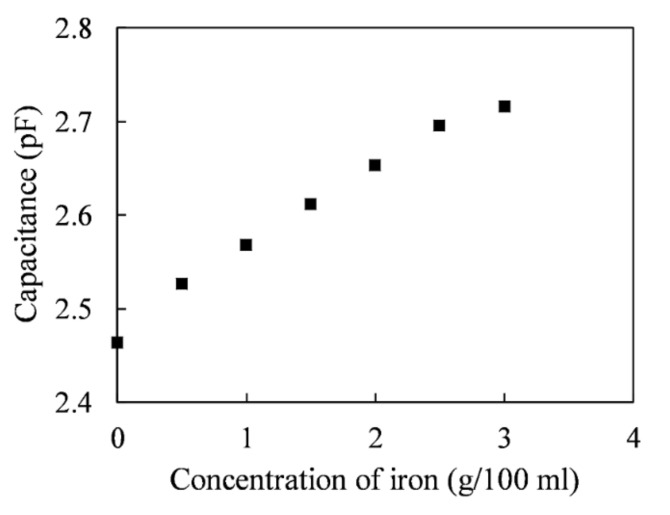
Measured capacitance increment of the iron particles.

The capacitance values of iron powders at different concentrations are plotted in Figure 6. The capacitances were measured by adding different weights of iron to 100 mL of EDM oil. When the iron concentration was 0.5 g/100 mL, the capacitance was 2.53 pF. When the iron concentration increased to 3 g/100 mL, the capacitance value increased to 2.72 pF. Therefore, the capacitance increment of the iron particles over the measured contamination concentration range was 0.087 pF per 1 g/100 mL.

### 5.3. Machining Measurements for EDM Oil

**Figure 7:**
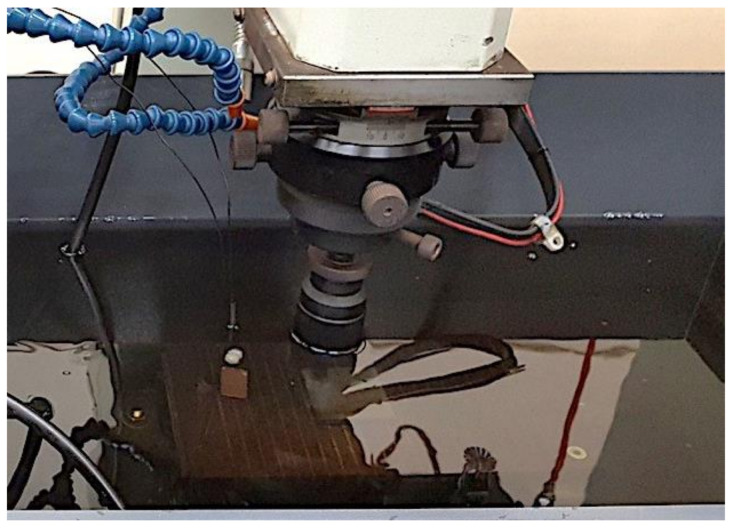
Photo of the EDM process.

**Figure 8:**
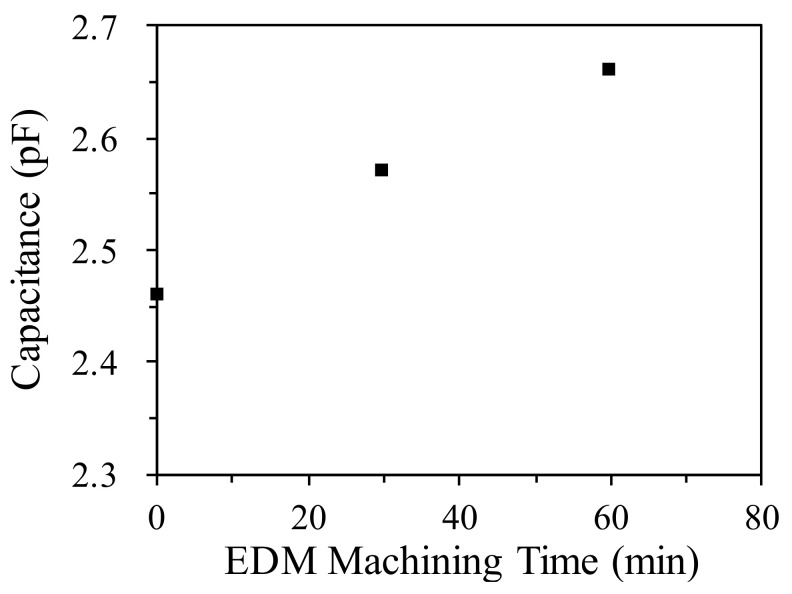
Measured capacitance increment for SS41 machining.

The change in the level of oil contamination during EDM was also measured in this study. SS41 low-carbon steel was processed in a processing tank, as shown in Figure 7. The capacitance value was recorded as processing time increased (Figure 8). The capacitance was positively correlated with the concentration of the processing oil. The capacitance of the pure processing oil before processing was 2.46 pF. After 60 min of machining, the equivalent capacitance of the oil increased to 2.66 pF. The equivalent capacitance of the sensor increased with the processing time.

### 5.4. Mixed Measurement and Analysis

The capacitance of the processing oil increased with the concentration of carbon black and iron particles in it. As previously seen, the increase rate of the capacitance with the concentration was higher for carbon black than for iron. To model the measurement data of the experiment, a linear polynomial regression was performed using the capacitance variation data measured in three material mixing experiments. The fitting equation and correlation coefficients of the two-dimensional variables are presented in Equation (7) and Table 1, respectively.

(7) $$Z=f\left(X,Y\right)=\alpha_{o}+\alpha_{CB}X+\alpha_{iron}Y,$$

**Figure 9:**
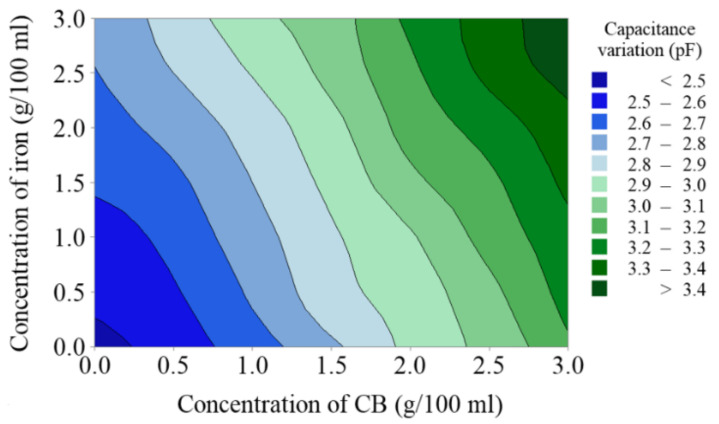
Contour of the measured capacitance increments versus the concentration of CB and Fe particles in EDM oil.

In Figure 9, the horizontal axis (X) represents the concentration of carbon black particles in grams per 100 mL of processing oil, the vertical axis (Y) represents the concentration of iron particles in grams per 100 mL of processing oil, and Z represents the capacitance increments. The color blocks of the contour map represent different capacitance measurement results. The linear fitting coefficients indicate that the rising trend of capacitance for CB particles is faster than that for iron particles.

**Figure 10:**
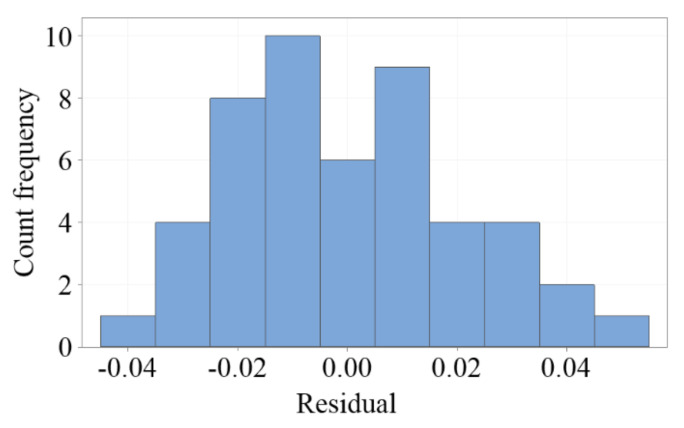
Histogram of the residuals, which are almost normally distributed.

**Figure 11:**
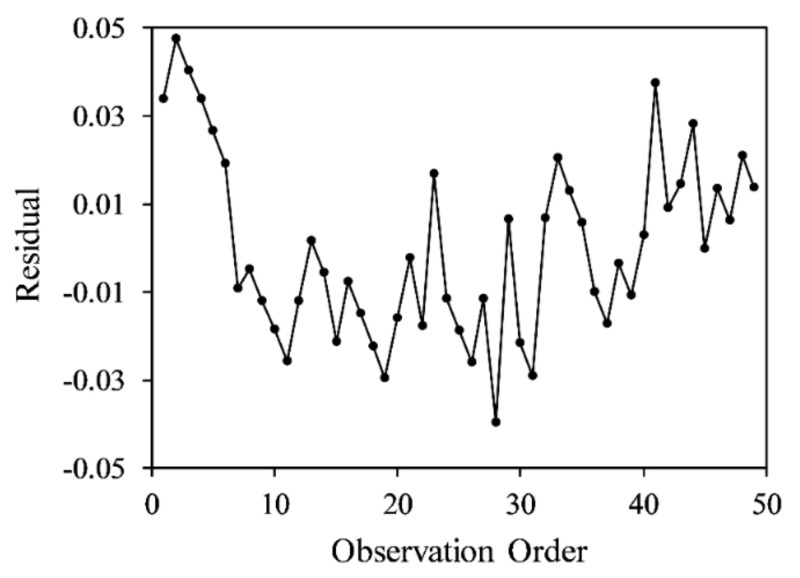
Sequence diagram illustrating that the residuals are not dependent on time. The residuals fluctuate randomly near 0.

A residual analysis was performed to determine the difference between the fitted predicted value and the actual measurement data. Figure 10 presents a histogram composed of the number of residual occurrences; it can be used to statistically observe whether the residuals have a normal distribution. Figure 11 plots the correlation between the residuals and the experimental order, which indicated that the residuals satisfied independent assumptions, and that the experimental results were not easily affected by time changes.

### 5.5. Prediction Results Obtained Using the EMT Model

**Figure 12:**
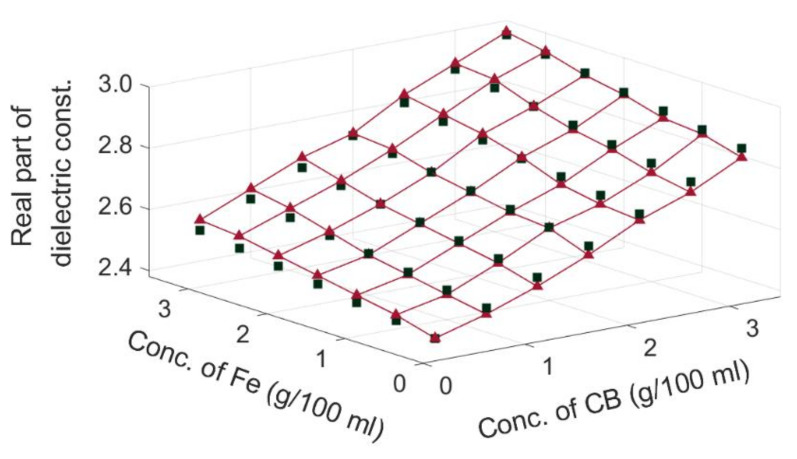
Comparison between the EMT results (green marks) and the experimental results (red marks) for the real part of equivalent relative permittivity.

To determine the EMT Model, we chose different β values and input them in Equation (6) to calculate the $\varepsilon_{eff}$. By comparing the numerical results with the experimental results, the β value with the least error was chosen. The closest match between the predicted and experimental results was obtained when β was −0.0769 in Equation (6). Because the imaginary part (${\varepsilon^{\prime \prime }}_{eff})$ of the equivalent relative complex permittivity cannot be measured by using the current conversion circuit directly, only the real part of the equivalent relative complex permittivity (${\varepsilon^{\prime }}_{eff})$ is represented on the vertical axis, as plotted in Figure 12. The green marks were obtained through EMT calculation and the red marks represented the experimental results obtained with the capacitive sensor. The results obtained with the EMT were in accordance with the experimental results.

In this EMT model, the relative complex permittivities of carbon black and iron are respectively represented as follows: $\varepsilon_{CB}\;≅\;-j\frac{3.5\times 10^{2}}{\omega \varepsilon_{0}}$ and $\varepsilon_{Iron}\;≅\;-j\frac{10^{7}}{\omega \varepsilon_{0}}$. The density of carbon black is 1.8 g/cm^3^ [25,26,27]. The complex dielectric constant of the pure processing oil was approximately 2.39. All the measurements were performed at f = 10 kHz and ε0≅ 8.85 × 10−12 Fm.

The RMSE (root-mean-squared error) between the predictions and measurements was 0.0209, i.e., less than 1% of the measurement results. As mentioned in the literature [18,19], the structure network formed by carbon black is complex, and different types of carbon black have different conductivities and densities. Therefore, this may affect the fitting parameter β of the EMT results.

## 6. Conclusions

In this paper, we assessed the quality of electrical discharge machining oil by analyzing variations in the equivalent relative permittivity of the capacitive sensor. The sensor has the advantages of a simple structure, DC voltage output, and instant measurement. In addition to using common processing materials, this study machined low-carbon steel (SS41) to measure the oil quality in terms of increased concentration of the impurities. A relationship between capacitance increments and processing time was obtained during EDM process. In addition, a set of experiments was performed in advance to build up a database. An equivalent medium theory model was then developed to predict the weight or volume percentages of the impurities in the mixed solution. Suitable fitting parameters were obtained to approximate the final experimental results.

## Figures and Tables

**Table 1:** The fitting coefficient.

Fitting Coefficient	The Meaning of Fitting Coefficient	Value
$\alpha_{o}$	The initial value of capacitance.	2.429
$\alpha_{CB}$	The rising trend of capacitance for CB particles	0.245
$\alpha_{iron}$	The rising trend of capacitance for iron particles	0.099
